# Notes from the Field: Ongoing Transmission of *Candida auris* in Health Care Facilities — United States, June 2016–May 2017

**DOI:** 10.15585/mmwr.mm6619a7

**Published:** 2017-05-19

**Authors:** Sharon Tsay, Rory M. Welsh, Eleanor H. Adams, Nancy A. Chow, Lalitha Gade, Elizabeth L. Berkow, Eugenie Poirot, Emily Lutterloh, Monica Quinn, Sudha Chaturvedi, Janna Kerins, Stephanie R. Black, Sarah K. Kemble, Patricia M. Barrett, MSD, Kerri Barton, D.J. Shannon, Kristy Bradley, Shawn R. Lockhart, Anastasia P. Litvintseva, Heather Moulton-Meissner, Alicia Shugart, Alex Kallen, Snigdha Vallabhaneni, Tom M. Chiller, Brendan R. Jackson

**Affiliations:** ^1^Division of Foodborne, Waterborne, and Environmental Diseases, National Center for Emerging and Zoonotic Infectious Diseases, CDC; ^2^Epidemic Intelligence Service, CDC; ^3^New York State Department of Health; ^4^Division of Epidemiology, New York City Department of Health and Mental Hygiene; ^5^School of Public Health, University at Albany, State University of New York; ^6^Chicago Department of Public Health; ^7^New Jersey Department of Health; ^8^Massachusetts Department of Public Health; ^9^Indiana State Department of Health; ^10^Oklahoma State Department of Health; ^11^Division of Healthcare Quality Promotion, National Center for Emerging and Zoonotic Infectious Diseases, CDC.

In June 2016, CDC released a clinical alert about the emerging, and often multidrug-resistant, fungus *Candida auris* and later reported the first seven U.S. cases of infection through August 2016 (*1*). Six of these cases occurred before the clinical alert and were retrospectively identified. As of May 12, 2017, a total of 77 U.S. clinical cases of *C. auris* had been reported to CDC from seven states: New York (53 cases), New Jersey (16), Illinois (four), Indiana (one), Maryland (one), Massachusetts (one), and Oklahoma (one) (Figure). All of these cases were identified through cultures taken as part of routine patient care (clinical cases). Screening of close contacts of these patients, primarily of patients on the same ward in health care facilities, identified an additional 45 patients with *C. auris* isolated from one or more body sites (screening cases), resulting in a total of 122 patients from whom *C. auris* has been isolated.

**FIGURE:**
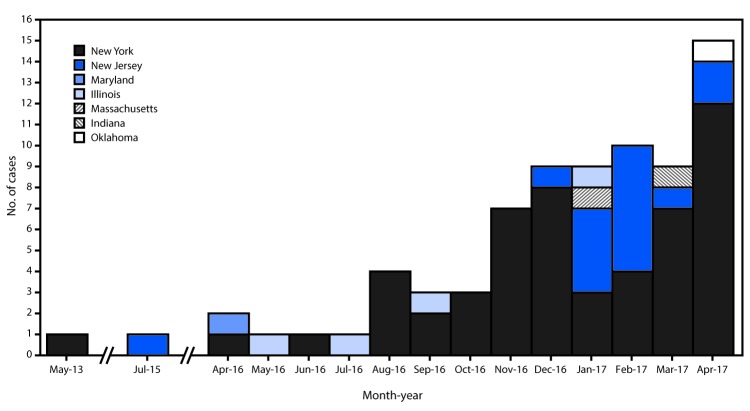
Number of health care–associated cases of *Candida auris* infection reported to CDC (N = 77) — seven states, May 2013–May 2017

Among the 77 clinical cases, median patient age was 70 years (range = 21–96 years), and 55% were male. *C. auris* was cultured from the following sites: blood (45 isolates), urine (11), respiratory tract (eight), bile fluid (four), wound (four), central venous catheter tip (two), bone (one), ear (one), and a jejunal biopsy (one). Antifungal susceptibility testing at CDC of the first 35 clinical isolates revealed that 30 (86%) isolates were resistant to fluconazole (minimum inhibitory concentration [MIC] >32), 15 (43%) were resistant to amphotericin B (MIC ≥2), and one (3%) was resistant to echinocandins (MIC >4). Most (69, 90%) clinical cases were identified in the New York City metropolitan area (53 in New York and 16 in New Jersey). Nearly all patients had multiple underlying medical conditions and extensive health care facility exposure. Epidemiologic links have been found between most cases. In Illinois, three cases were associated with the same long-term care facility. In New York and New Jersey, cases were identified in multiple acute care hospitals, but further investigation found most had overlapping stays at interconnected long-term care facilities and acute care hospitals within a limited geographic area. The case in Massachusetts was linked to the Illinois cases. The cases in Indiana and Oklahoma occurred in patients who had recently received health care in other countries.

Testing for *C. auris* colonization, using a composite swab of the groin and axilla, was conducted for 390 close contacts of the 77 patients in three states, primarily patients on the same ward in health care facilities because of the risk for environmental contamination and transmission from health care personnel. The two body sites tested were selected based on results of previous investigations. Forty-five (12%) colonized persons were identified (24 in New Jersey, 17 in New York, and four in Illinois). Contact Precautions were recommended for colonized patients in health care facilities. Nasal swabs also were collected from 184 (47%) contacts; two swabs (1%) were positive, both from patients with positive groin/axilla swabs. Environmental testing of patients’ rooms identified *C. auris* from mattresses, beds, windowsills, chairs, infusion pumps, and countertops, indicating *C. auris* environmental contamination. *C. auris* was not isolated from rooms after thorough cleaning with a sodium hypochlorite–based disinfectant.

All *C. auris* isolates were forwarded to CDC for whole-genome sequencing and comparison with previously sequenced international isolates, which clustered into four distinct clades (*2*). Isolates from within each state were highly related. New York isolates, with the exception of one clinical and one screening case, were highly related to one another and grouped in the same clade as isolates from South Asia. Isolates in New Jersey also were similar to those from South Asia but were distinct from those in New York. Illinois isolates were nearly identical to one another and grouped with isolates from South America. These data suggest multiple introductions of *C. auris* into the United States followed by local transmission.

Ongoing investigation of U.S. *C. auris* cases provides epidemiologic and laboratory data suggesting that this fungus can spread within health care facilities and that interventions are needed to prevent transmission during this early stage of *C. auris* emergence. As of May 2017, recognized U.S. *C. auris* cases were concentrated in health care facilities in three separate geographic areas, and most cases were in chronically ill patients with long stays at high-acuity skilled nursing facilities (e.g., facilities providing mechanical ventilation). Apart from one case identified in 2013, clinical laboratories serving health care facilities with *C. auris* cases have not identified suspected *C. auris* isolates from before 2015 from retrospective microbiology record reviews, suggesting recent *C. auris* emergence in those locations. However, the disease might exist elsewhere, because some laboratories do not fully characterize *Candida* species or are otherwise unable to detect *C. auris*.

CDC has worked with state and local partners to develop and share infection control recommendations to help curb the spread of *C. auris* (*3*). Current recommendations for *C. auris*–colonized or infected patients include 1) use of Standard Precautions and Contact Precautions, 2) housing the patient in a private room, 3) daily and terminal cleaning of a patient’s room with a disinfectant active against *Clostridium difficile* spores (an update from previous disinfectant recommendations) (*4*), and 4) notification of receiving health care facilities when a patient with *C. auris* colonization or infection is transferred. Accurate identification of *C. auris* and adherence to infection control practices, coupled with ongoing public health surveillance and investigations, are needed to halt the spread of *C. auris* in the United States.

## References

[1] Vallabhaneni S, Kallen A, Tsay S, Investigation of the first seven reported cases of *Candida auris*, a globally emerging invasive, multidrug-resistant fungus—United States, May 2013–August 2016. MMWR Morb Mortal Wkly Rep 2016;65:1234–7. 10.15585/mmwr.mm6544e1 27832049

[2] Lockhart SR, Etienne KA, Vallabhaneni S, Simultaneous emergence of multidrug-resistant *Candida auris* on 3 continents confirmed by whole-genome sequencing and epidemiological analyses. Clin Infect Dis 2017;64:134–40. 10.1093/cid/ciw691 27988485 PMC5215215

[3] CDC. *Candida auris* interim recommendations for healthcare facilities and laboratories. Atlanta, GA: US Department of Health and Human Services, CDC; 2017. https://www.cdc.gov/fungal/diseases/candidiasis/recommendations.html#infection

[4] Environmental Protection Agency. Pesticide registration. List K: EPA’s registered antimicrobial products effective against *Clostridium difficile* spores. Washington, DC: Environmental Protection Agency; 2017. https://www.epa.gov/pesticide-registration/list-k-epas-registered-antimicrobial-products-effective-against-clostridium

